# Serological investigation of visceral *Leishmania* infection in human and its associated risk factors in Welkait District, Western Tigray, Ethiopia

**DOI:** 10.1016/j.parepi.2017.10.004

**Published:** 2017-11-04

**Authors:** Abrha Bsrat, Mebrahtu Berhe, Endalemaw Gadissa, Habtamu Taddele, Yohannes Tekle, Yohannes Hagos, Adugna Abera, Messele G/micael, Tehetna Alemayhu, Getachew Gugsa, Abraham Aseffa

**Affiliations:** a Mekelle University College of Veterinary Medicine, Mekelle, Ethiopia; b Veterinary Drug and Feed Administration and Control Authority, Tigray, Ethiopia; c Armauer Hansen Research Institute, Addis Ababa, Ethiopia; d Wollo University College of Veterinary Medicine, Dessie, Ethiopia

**Keywords:** Ethiopia, Human, Leishmanin skin test, Risk factors, Sero-prevalence, Visceral leishmaniasis, Welkait

## Abstract

Visceral leishmaniasis (VL) is major neglected public health problem in terms of geographical spread and incidence in Ethiopia. Magnitude, public health impact and dynamics of VL were not well studied in Welkait District, Western Tigray, though the area is known for VL. Hence, this study aimed to determine sero-prevalence of human VL and associated risk factors in Welkait as new foci. A cross sectional study design was employed in this study. Two stage stratified random sampling method was used to select study participants. Hence, a total of 329 human study participants were included for serological survey using ITleish and leishmanin skin tests. Semi structured questionnaire was also used to identify VL associated risk factors. Univariate and multivariate logistic regression statistical methods were used to determine the degree of association. The overall sero-prevalence of human VL in the study area was found to be 8.81%. Statistical significant difference in the prevalence of the disease was found among Sub-districts, sex, re-settlement, sleeping outdoor and dog ownership (*P* < 0.05). Participants who resettled from their original place were found 2 times (AOR = 2.143; 95% CI = 1.02, 14.20) more vulnerable to VL infection. Those who had an experience of sleeping outdoor were found almost 4 times (AOR = 4.29; 95% CI = 1.58, 11.69) more likely to be at risk of acquiring VL infection than those sleep indoor. Furthermore, individuals who owned dogs were 3 times more prone to the VL infection than their counterparts (AOR = 3.37; 95% CI = 1.29, 8.76). Alarming sero-positivity of human VL was recorded from new foci. Hence, it is recommended to improve the VL health services in the study area. The investigation also invites further study on VL dynamics in the study area.

## 1 Background

Leishmaniasis that has a global distribution is among the neglected parasitic disease of the tropics (World Health Organization (WHO), 2015). After malaria, leishmaniasis is the second most important vector-borne diseases accounting for an estimated 50,000 deaths per annum (World Health Organization (WHO), 2015). The disease is caused by obligate intracellular protozoa of the genus *Leishmania*. It infects numerous mammalian species including humans. Leishmaniasis is transmitted by the bite of infected female sand flies of the genera *Phlebotomus* and *Lutzomyia* in the Old and the New World respectively (Gramiccia, and Gradoni, 2005). Leishmaniasis is a group of diseases with diverse epidemiological and clinical patterns (from self-healing skin ulcers to severe, life threatening visceral disease (VL)) (World Health Organization (WHO), 1996; Dantas-Torres, 2009).

VL infection is prevalent in the tropical and subtropical regions of 79 countries (Alvar et al., 2006, Alvar et al., 2012). Approximately 300,000 new cases of VL occur annually (90% in Bangladesh, Brazil, Ethiopia, India, South Sudan and Sudan). The annual estimated number of deaths from VL ranges from 20,000–50,000 (World Health Organization (WHO), 1996; Alvar et al., 2012). The geographical distribution of the disease can depend on environmental changes (climate change, deforestation and urbanization), population movements between endemic and non-endemic zones, appearance of therapy-resistant strains and immune suppression, mainly due to malnutrition and co-infection with the human immunodeficiency virus (HIV) (World Health Organization (WHO), 1996; Dantas-Torres, 2009, Alvar et al., 2006, Thakur et al., 2000).

As in other East Africa countries, VL (also called “kala-azar”) caused by *L. donovani* (Marlet et al., 2003, Basiye et al., 2010) with a principal vector of *P. orientalis* (Gebre-Michael et al., 2010) is endemic in Ethiopia, with a patchy distribution in the southern and northwestern lowlands (Hailu et al., 2006). According to the recent report of World Health Organization (WHO) (2017), Ethiopia has reported the third largest number of VL cases (1990) following South Sudan (2840) and Sudan (2813) of any country in sub-Saharan Africa region. Human VL caused by *L. donovani* is endemic in the northwestern lowlands around Humera and Metema with an incidence of 1000–2000 cases annually (Ritmeijer et al., 2001, Ritmeijer et al., 2006). Reports from domestic animals like dogs have been also indicated in the area for *L. donovani* (Kalayou et al., 2010).

Even though several studies showed that VL is endemic in Ethiopia, there is limited published data about the status of the disease other than hospital reports in the study area, Welkait. The study area has been identified by the government as one of the ten newly under construction sugar mega projects in the country. Hence, habitants of the surroundings have been re-collected and re-settled on specific areas in the form of town (Central Statistics Agency (CSA), 2013) leading to new urbanizations. This in turn brings new dynamism of increasing risk factors which include rapid urbanization, deforestation, new settlements, migration, cross-border movement and agricultural development making VL a growing public health concerns in the study area. Moreover, dogs which are reported to harbor *L. donovani* (VL causing pathogen), share the same living shelter with their owners (Kalayou et al., 2010). The area is also endowed with suitable ago-ecological conditions for sand fly multiplication. However, no scientific work has been done about the dynamics of VL in the Welkait district. Therefore, this study was aimed to determine the sero-prevalence of VL in human and identify the associated risk factors which in turn contribute for designing and implementations of future VL prevention and control programs.

## 2 Materials and methods

### 2.1 Description of the study area

**Fig. 1:**
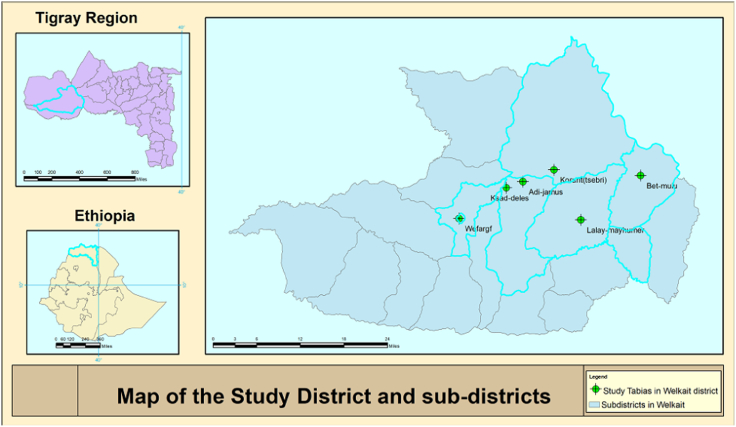
Map of the study area, Welkait District, Western Tigray, Ethiopia.

The study was conducted in Welkait district, Western zone, Tigray region, Ethiopia. Welkait is located 437 km west and 1220 km northwest far from the capital city of Tigray region (Mekelle) and country capital city (Addis Ababa), respectively at 13°30′00″ and 14°07′00″ North latitude, and 36°40′15″ and 37°48′00″ East longitude with an altitude ranges from 677 to 2755 m above sea level. The district has 28 sub-districts of which 14 are with lowland agro-ecology (Fig. 1). The annual temperature and unimodal rainfall of the district are 17.5–25 °C and 700–1800 mm, respectively (Office of Plan and Finance Welkait district, 2015). Six sub-districts namely: WefArgif, KisadDelesa, AdiJamus, Korarit, BetMulu and LaelayMayhumer were selected purposively. The total population of the district settled at 35,215 household is 163,993 (83,129 male and 80,110 females) of which 138,731 are rural settlers (Central Statistics Agency (CSA), 2013). According to the Welkait district Agricultural and rural development offices data record and annual Report, the district has 1600 owned-dogs (excluding stray dogs).

### 2.2 Study design and sample size determination

A cross sectional study design was employed to determine the sero-prevalence of VL in human and identify the associated risk factors. Sample size was calculated as per Thrusfield (2005) considering 30% previous prevalence of VL (Alvar et al., 2007) (considering the geographical location both found on north west Ethiopia and could have interaction line following the way to Sudan from Bahirdar), at 95% level of confidence, 5% desired precision. Accordingly, a total of 329 human sera were collected.

### 2.3 Sampling method

Samples were selected using two stage stratified random sampling. First stage, six sub-districts were selected from the 28 sub-districts in Welkait purposively based up on their previous history of exposure to VL using the Welkait-district health office annual reports recorded data, physical geographical situation and transport accessibility. A complete household listing was then carried out in each selected sub-districts and a proportionate sample size allocation was done to each selected sub-districts. Second stage, households (329) was randomly selected from the six selected sub-districts. One participant was then randomly selected from each household and contacted for informed consent/assent. Semi structured questioner was administered to obtain required associated risk factors. Beside to this, selected study participants were asked for owning of dogs. Accordingly, from 81 participants who were identified to own dogs, 46 were volunteer for blood samples acquisition from their dogs.

### 2.4 Sample collection and diagnosis

**Fig. 2:**
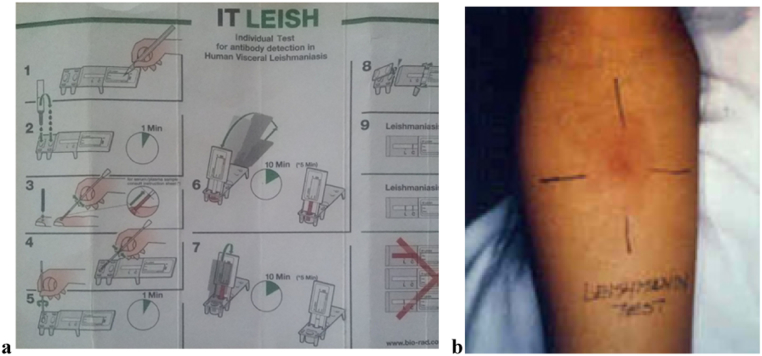
Serological tests for visceral Leishmaniasis, basic procedure a) ITleish (It-Leish brochure), and b) leishmanin skin test (http://www.who.int/leishmaniasis/surveillance/slides_manual/en/index11.html).

A total of 329 human blood samples and 46 dog blood samples were collected using plastic capillary tube of the ITleish kit from finger prick (human) and disposable syringe from cephalic/saphenous vein (dogs). Furthermore, leishmanin skin test (LST; Pasteur Institute of Iran, Tehran, Iran) was also conducted on 306 human participants who were tested for ITleish following the manufacturer's instruction. However, no skin test was done for dogs. Both techniques were carried out by trained clinical nurse. The two antibody detection tests, the ITleish rapid diagnostic test (RDT) (DiaMed-IT LEISH rK39 (DiaMed AG, Cressier sur Morat, Switzerland) (Benson et al., 1996, Mettler et al., 2005, Abera et al., 2016) (Fig. 2a), which referred to rk39 antigen based immunochromatographic test (human and dog) and the leishmanin skin test (human) (hypersensitivity reaction based test; Pasteur Institute of Iran, Tehran, Iran) (Ali et al., 2004, Abera et al., 2016) (Fig. 2b) were used for the serological investigation of VL.

### 2.5 Data analysis

Collected data were entered into an excel sheet and analyzed using STATA 11.0 statistical software. Descriptive statistics were employed to summarize the data and expressed in terms of frequencies and percentages. Univariate and multivariate logistic regression statistical methods were used to determine the association of the disease with the risk factors and expressed as odds ratio and 95% confidence interval. For all analysis a *P* < 0.05 was considered for significance difference.

### 2.6 Ethical consideration

Ethical clearance approval to conduct the study was obtained from Institutional Review Board of Mekelle University, College of Veterinary Medicine (dogs) and College of Health Sciences (human). Furthermore, verbal informed consent/assent was obtained from all selected individuals for the study after explaining the purpose of the study in the local language, Tigrigna.

## 3 Results

### 3.1 Prevalence of human visceral leishmaniasis

**Fig. 3:**
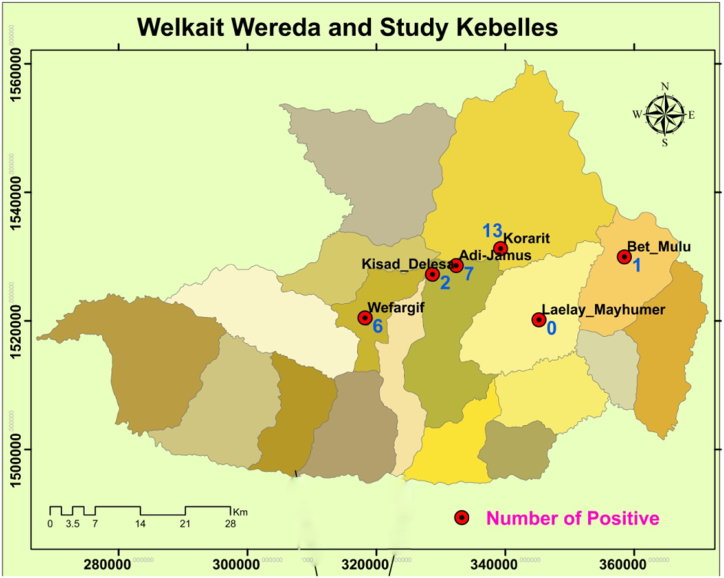
Distribution of visceral *Leishmania* infection in the study areas.

Among the 329 serologically examined human participants, 29 (8.81%) were found sero-reactive for ITleish RDT. Furthermore, among the 306 human participants tested for skin test, 18(5.88%) were found reactive. The number of reactive individuals in the respective 6 study sub-districts is also given below (Fig. 3).

### 3.2 Association of risk factors with occurrence of human VL infection

Statistical significant difference in the prevalence of VL were found among sub-districts, sexes, re-settlement status, sleeping outside and dog ownership (*P* < 0.05), whereas variables among age, occupation, presence of black soil, presence of *Acacia seyal*/*Balanite aegyptiaca* tree, use bed net were found to be statistically insignificant in the final model (Adjusted odds ratio) (Table 1).

**Table 1:** Association of risk factors with the occurrence of human VL.

Predictor variable		Number examined	Number positive N (%)	COR (95%CI)	*P*-value	AOR (95%CI)	*P*-value
Sub-districts	Korarit	74	13 (17.57)	8.52 (1.728, 67.737)	0.0277	7.719 (1.120, 72.696)	0.020
	WefArgif	61	6 (9.84)	4.36 (7.505, 37.680)	0.0490	2.328 (0.249, 21.816)	0.459
	KsadDelesa	48	2 (4.14)	1.74 (0.152, 19.905)	0.1801	1.079 (0.082, 14.287)	0.954
	AdiJamus	56	7 (12.50)	5.71 (10.675, 48.399)	0.0356	3.976 (0.232, 68.173)	0.074
	BetMulu	49	1 (2.04)	1 (Reference)	–	1 (Reference)	–
	LaelayMayhumer	41	0 (0.00)	–	–	–	–
Sex	Male	211	27 (12.80)	8.51 (1.986, 36.464)	0.004	7.718 (1.633, 36.481)	0.010
	Female	118	2 (1.69)	1 (Reference)	–	1 (Reference)	–
Age group (year)	< 15	67	8 (11.94)	3.19 (0.646, 15.722)	0.155	–	–
	15–30	95	11 (9.32)	2.42 (0.515, 11.328)	0.263	–	–
	30–50	118	8 (8.42)	2.16 (0.441, 10.589)	0.342	–	–
	> 50	49	2 (4.08)	1 (Reference)	–	–	–
Occupation	Farmer	212	20(9.43)	2.60 (0.335, 20.252)	0.360	–	–
	Student	86	8(9.30)	2.56 (0.306, 21.515)	0.386	–	–
	Employee	26	1(3.85)	1 (Reference)	–	–	–
	Daily laborer	5	0(0.00)	–	–	–	–
Re-settlement status	Yes	92	15(16.30)	3.10 (1.832, 6.722)	0.0046	2.143 (1.023, 14.204)	0.034
	No	237	14(5.91)	1 (Reference)	–	1 (Reference)	–
Presence of black soil	Yes	246	30(12.21)	1.64 (0.728, 3.679)	0.244	–	–
	No	83	6(7.23)	1 (Reference)	–	–	–
Presence of *Acacia seyal*/*Balanite aegyptiaca* tree	Yes	162	18 (11.11)	1.77 (0.809, 3.881)	0.146	–	–
	No	167	11 (6.59)	1 (Reference)	–	–	–
Sleeping outside	Yes	155	23 (14.84)	4.88 (1.931, 12.328)	0.0002	4.292 (1.576, 11.693)	0.004
	No	174	6 (3.45)	1 (Reference)	–	1 (Reference)	–
Use of bed net	Yes	267	21 (7.61)	0.46 (0.193, 1.110)	0.1000	–	–
	No	53	8 (15.09)	1 (Reference)	–	–	–
Dog ownership	Yes	81	14 (17.28)	3.25 (1.492, 7.062)	0.0037	3.367 (1.294, 8.762)	0.013
	No	248	15 (6.05)	1 (Reference)	–	1 (Reference)	–

Highest prevalence (17.57%) was found from Korarit sub-district followed by AdiJamus sub-district (12.50%) with zero VL prevalence in LaelayMayhumer. Being a resident of Korarit sub-district increases the odds of getting VL infection by nearly 8 times (AOR = 7.72; 95%CI = 1.12, 72.70) than living in BetMulu after adjusting for sex, re-settlement, sleeping outside and dog ownership. Male participants were found to be highly infected (12.80%). Male participants were found 8-fold (AOR = 7.72; 95% CI = 1.63, 36.48) exposed to the disease compared to that of females. Participants which had involved in the re-settlement program following the construction of the mega sugar industry project in the study area had found nearly 2 times (AOR = 2.143; 95% CI = 1.02, 14.20) more vulnerable to VL infection. Higher prevalence of the disease was found among individuals who had the habit of sleeping outdoor (14.84%). Participants who had an experience of sleeping outdoor were also found almost 4 times (AOR = 4.29; 95% CI = 1.58, 11.69) more likely to be at risk of acquiring VL infection than those sleep indoor. Those who owned dogs were found to have high prevalence of VL (17.28%) as compare to those who did not have dogs. Individuals who owned dogs were 3 times more prone to the VL infection than their counterparts (AOR = 3.37; 95% CI = 1.29, 8.76) after adjusting for the other variables (Table 1).

Among the total 329 serologically tested participants, 81 individuals were dog owners and of which 46 of them were volunteers to give blood samples from their dogs. A serological study was then carried out on their dogs parallel with their owners using ITleish RDT. Accordingly, 4.35% (2/46) of dogs and 13.04% (6/46) of dog owners were found positive for ITleish RDT and surprisingly, owners of those 2 positive dogs were found positive to ITleish RDT (Table 2).

**Table 2:** Describing the sero-positivity of VL on dogs and their owners, using ITleish RDT.

Residence area	Examined dogs & humans	No. of positive dogs	No. of positive humans
WefArgif	2	0	0
KsadDelesa	14	**1**	**1**
AdiJamus	2	**1**	**1**
Korarit	9	0	3
LaelayMayhumer	7	0	0
BetMulu	12	0	1
**Total**	**46**	**2**	**6**

## 4 Discussion

The current study revealed an overall prevalence of human VL 8.81%. The current finding was found higher than previous reports 1.02% sero-prevalence (Sordo et al., 2009) from Libokemkem area of the Northwestern Ethiopia. Possible reason for this variation could be due to difference in agro-ecological settings, presence of possible predisposing risk factors and progressive intervention activities made following the outbreak of 2005 in Libokemkem. The case in Libokemkem was report done years after active epidemic outbreak but the current record was assessment to look sero-prevalence reactivity with unknown scientific presence in a new expected area. Accordingly, the 8.81% overall VL prevalence of this study indicated existence of the disease in the district that could have short or long time interval. On the other hand the district is neighboring to Kaft-Humera, an endemic district in the region (Kalayou et al., 2010), which might share VL infection considering the agro-ecological similarity for VL dynamics. However, leishmanin skin test finding of the current study (5.88%) showed lower than previous reports (Ali et al., 2004, Ali et al., 2002), with a positive reaction of 40% in middle and lower Awash valley of Ethiopia. This variation can be explained by difference in sample size, research methodology and endemicity of VL in the study areas.

Residence area, sex, resettlement status, sleeping outdoors and dog ownership were found statistically significant with the occurrence of VL in the current study. Higher prevalence of the disease was found in Korarit sub-district than the other sub-districts. This significant variation could be associated with the re-settlement program in which Korarit residents are migrated from their original settlement, Welkait sugar project development site, which could increase the chance of exposure to possible vectors. In line with this finding, Dawit et al. (2013) reported that, migration was an important predictor of VL infection. The movement of non-immunized peoples in to an area with existing transmission cycles would elevate the risk of developing VL infection. Welkait district is neighboring to Humera, VL endemic district, in Tigray (Kalayou et al., 2010). Sharing of labors during harvesting time is very common. Sandflies are common in Welkait which can be infected from those coming from Humera. The communities in the district were habituated separately. However, due to the sugar project, they resettled to aggregated areas that can expose them to those migrated individuals from endemic area and the sandflies too. Hence, endemicity can be created in the area. Second possible reason could be its previous unknown status in the distinct. The disease could exist undetected in the district but expressed after resettlement.

The prevalence of VL at WefArgif sub-district, with midland agro-ecology (2040 m a.s.l.), was found to be higher than the other three low land sub-districts in which the prevalence of VL from higher altitude is not expected due to the vector ecology. This could be explained by the vector might adapted to higher altitude due to climate change, movement of residents to nearby lowlands for farming with low protective measures due to lack of awareness.

Males were found with highest risk for VL infection than females. This finding is in line with a report from north Gonder (Bantie et al., 2014) indicating higher risk of VL infection on males. The variation in the prevalence might be due to exposure to sand fly where males are mostly engaged in outdoor activities such as crop harvesting, sleeping outdoor to keep their farm and animals from theft. Besides, in this study individuals who use outdoor sleeping were found with higher risk for VL infection. Similar findings were recorded by (Bantie et al., 2014, Bashaye et al., 2009, Malaria Consortium, 2010).

Individuals who owned dogs were found to be at high risk of developing the disease compare to their counterpart in the current study. This finding is in agreement with findings reported from various areas of Ethiopia (Bantie et al., 2014, Bashaye et al., 2009, Belo et al., 2013, Yared et al., 2014). Possible reason for this could be due to the fact that, dogs might have a role for VL distribution in the area although dogs are not incremented as reservoir host for VL in Ethiopia (Bashaye et al., 2009, Dereure et al., 2000, Hassan et al., 2009).

Even though the prevalence of VL among participants who use bed net and who do not is statistically insignificant, higher proportion of the diseases was observed among participants who did not use bed net in the current study. This finding is supported by other findings from Ethiopia (Bantie et al., 2014, Bashaye et al., 2009). Furthermore, in the current study, presence of black soil and *Acacia seyal*/*Balanite aegyptiaca* trees with occurrence of the diseases was found to be statistically insignificant despite higher prevalence was found in those areas that have black soil and *Acacia seyal*/*Balanite aegyptiaca* trees. Hence, further study seems crucial to see their role on VL dynamicity in the area since they are known habitats of *Phlebotomus orientalis*, already reported as endemic in neighboring district Humera (Lemma et al., 2014).

## 5 Conclusion

Sero-reactive human subjects for VL were recorded in the current study. Residence, sex, re-settlement status, outdoor sleeping and dog ownership have found as potential risk factors for VL distribution in the study district. Further investigation on transmission dynamics of VL circulation considering sand fly, *Leishmania* parasite and the role of dogs in the district is vital. Effective and well targeted control measures should be developed with a special concern on the identified risk factors.

## Competing interest

The authors declare that they have no competing interests.

## Authors' contributions

All authors read and approved the final version of the manuscript. AB, MB, EG, HT, YT, YH, AA, MG, GG, TA, and AA conceived the study. AB generated the idea, performed field and laboratory experiments, analyzed the data, dissemination, and prepared the paper. MB and HT performed enrichment of the idea, field and laboratory experiments, data analyze, dissemination and manuscript write up. YT and MG carried out data collection and diagnosis. EG, AA and AA kit support and training for field professionals. YH participated on proposal development, data analyze, dissemination and manuscript write up, GG and TA have done data entry, processing, dissemination and reporting.

## Appendix A Supplementary data

Supplementary material Image 1
