# Supplementary material for: Serological investigation of visceral Leishmania infection in human and its associated risk factors in Welkait District, Western Tigray, Ethiopia
Source: Parasite Epidemiol Control. 2017 Nov 4;3(1):13–20. doi: 10.1016/j.parepi.2017.10.004 (PMC5952676; doi:10.1016/j.parepi.2017.10.004)
Supplement: Supplementary file 1 — Supplementary material [file mmc1.doc]

# Annexes

Annex I: Pictures of possible sand fly habitats observed in Welkait District, Western Tigray


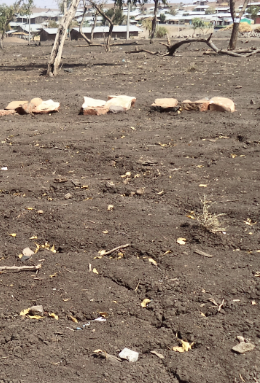

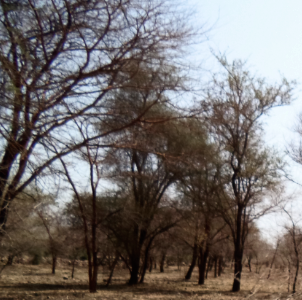


a, cracked black cotton **clay-soil** b, ***Acacia seyal*** -tree (local name:che'a)


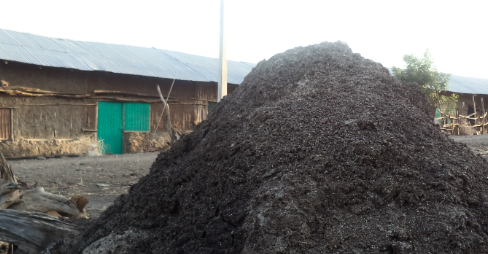

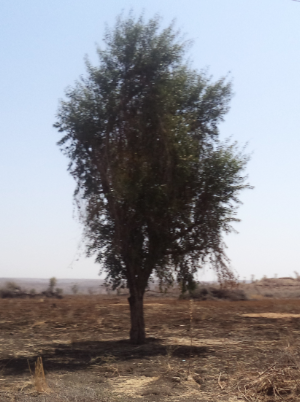


a, **Termite-hills** near a living house (lacal name: gulla) b, ***Balanites aegyptiaca*** -tree(lacal name: meqie)


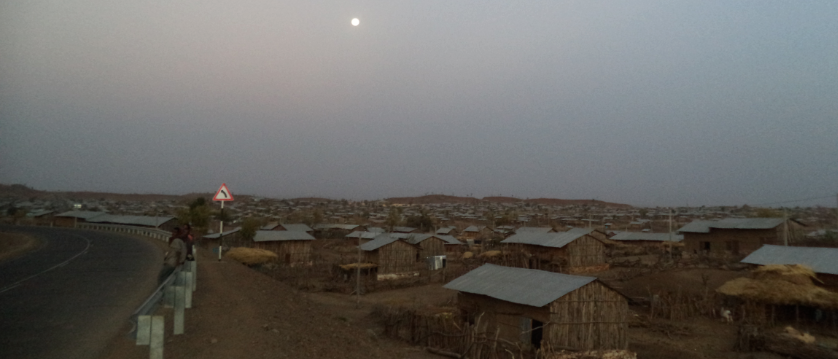


**Korarit** : one of the resettlement program established sub-districts of Welkait dstrict
